# Surgical factors affecting oculocardiac reflex during strabismus surgery

**DOI:** 10.1186/s12886-018-0771-9

**Published:** 2018-04-19

**Authors:** Suk-Gyu Ha, Jungah Huh, Bo-Ram Lee, Seung-Hyun Kim

**Affiliations:** 1 Department of Ophthalmology, Korea University College of Medicine, Seoul, Korea; 2 Department of Ophthalmology, Korea University Anam Hospital, 73, Inchon-ro, Seongbuk-gu, Seoul, 02841 South Korea

**Keywords:** Oculocardiac reflex, Strabismus, Surgery

## Abstract

**Background:**

To investigate surgical factors associated with the occurrence of oculocardiac reflex (OCR) and changes in heart rate (HR) during strabismus surgery.

**Methods:**

Patients who underwent strabismus surgery under general anesthesia were enrolled in this study. The HR during surgery was measured at baseline, and at the following points during surgery: traction of the muscle, maximal increase after traction (adrenergic phase), and the cutting of the muscle. OCR was defined as an HR reduction of more than 20% at traction of the muscle, when compared to baseline HR. The HR at each stage during the surgery was compared between patients with and without OCR.

**Results:**

A total of 162 operated muscles from 99 patients were enrolled. The incidence of OCR was 65% in patients. In patients with two muscle surgeries, there were significantly more OCRs in the first operated muscle than in the second operated muscle (*p* < 0.01). The difference in the decrease in HR in patients with OCR was significantly lower than that in patients without OCR at traction of the muscle, the adrenergic phase, and the cutting of the muscle (all, *p* < 0.01). The first operated muscle was a significant risk factor associated with the occurrence of OCR (OR = 3.95, *p* < 0.01).

**Conclusion:**

The first operated muscle in patients with two muscle surgeries was a significant risk factor for OCR. Decreased HR at the traction of the muscle during surgery did not fully recover in patients with OCR.

## Background

Oculocardiac reflex (OCR) is a phenomenon defined by bradycardia or dysrhythmia during strabismus surgery [1]. Although the definition of OCR has not yet been formally established, prevalence of OCR ranges from 14% to 90%, based on varying published definitions [2, 3]. Oculocardiac reflex is commonly caused by the traction on the extraocular muscle (EOM), which, through the ophthalmic branch of trigeminal nerve, stimulates the vagal center. The afferent arm of the reflex is the ophthalmic branch of the trigeminal nerve, and the efferent arm is the vagus nerve, which diminishes sinoatrial node impulses and leads to bradycardia [1, 4]. Transient cardiac arrest has been reported to occur in about 1/2200 cases during strabismus surgery [5]. Sino-atrial arrests and ventricular fibrillation have been reported [6, 7].

Previous studies have investigated the effect of anesthetic medication on the occurrence of OCR. To reduce OCR, retrobulbar block and premedication with anticholinergics have been applied. [3, 8–10] In spite of these attempts, methods of preventing OCR have not yet been found to be consistently effective. In addition, there are only a few studies that evaluate the relationship between strabismus surgery and OCR [11, 12].

The OCR commonly occurs following the traction of the EOM. Routine strabismus surgery requires 100–200 g tension on rectus muscles. Deliberately gentle surgery may take 50 g tension [13]. Braun et al. showed that sustained traction of 600 g of the EOM induced a counter-regulatory effect against OCR and increased the heart rate (HR) following the occurrence of OCR [14]. This phenomenon was referred to as the adrenergic phase of OCR. During strabismus surgery, several manipulations of the EOM, such as traction, re-traction, and cutting, are performed on the same muscle. Thus, stimulation of the EOM may be repeated and may vary, depending on the surgical method and other surgical factors, during the strabismus surgery.

The aim of this study is to investigate surgical factors associated with the occurrence of OCR and changes of HR in patients with OCR during strabismus surgery.

## Methods

The study protocol was approved by the Korea University Anam Hospital Institutional Review Board and adhered to the tenets of the Declaration of Helsinki. The medical charts of the patients who had undergone strabismus surgery under general anesthesia, at Korea University Anam Hospital, Seoul, Korea, between October 2015 and January 2016, were reviewed retrospectively. Patients classified as American Society of Anesthesiologists class I or II were selected. Patients were excluded if they had a history of bronchial asthma, congenital heart disease, central nervous disease, or other organic dysfunction. In pediatric patients under 18 years of age, intravenous atropine (0.01 mg/kg) was administered as a premedication and all patients were instructed not to eat or drink for 8 h prior to surgery. During the operation, thiopentone (5 mg/kg) and rocuronium (0.6 mg/kg) were administered to facilitate endotracheal intubation. Anesthesia was maintained with sevoflurane, an inhalational anesthetic. The HR was monitored every 5 s during anesthesia.

Recession or resection surgery of the EOM were performed based on their diagnosis. One single surgeon (SHK) performed all of the surgeries. Under general anesthesia, a fornix incision of conjunctiva was made. The EOM was engaged with a muscle hook and completely exposed with traction. The intramuscular septum was incised. In recession surgery, a suture was passed 1 mm from the insertion on the tendon and a locking bite suture was done in the superior and inferior margin of the muscle. The tendon was cut from the sclera. The EOM was reattached to the sclera according to the surgical amount. In resection surgeries, a suture was made at the superior and inferior margin of the muscle apart from the insertion according to the surgical amount. Though a tension gauge was not employed, the surgeon manipulated the extraocular muscle gently with uniformed tension in each of procedures. For recession of the inferior oblique muscle, following the isolation of the muscle, posterior fibers of the inferior oblique muscle were bunched using a single suture, toward the main muscle belly. The isolated inferior oblique muscle was placed next to vortex vein, behind the inferior rectus muscle. Based on the preoperative angle of deviation in patients, recession of the ipsilateral superior rectus muscles was performed in combination. The procedure of inferior oblique recession was similar to that for recession rectus muscle surgery. The surgical amount was determined by the patient’s angle of deviation, according to standardized values. The conjunctiva were closed with interrupted sutures at two points.

The data, collected preoperatively, included information regarding age at surgery, gender, and type of strabismus. During strabismus surgery, the number and sequence of the operated muscles, HR before traction of the EOM (baseline HR), maximum decreased HR after traction of the EOM, HR at maximum recovery from decreased HR and maintained during traction of the EOM (adrenergic HR), and HR at the cutting of the muscle, were collected. Oculocardiac reflex was defined as a decrease in HR greater than or equal to 20% rather than maximum at first traction of the muscle.

If a decrease of HR was noted during traction, we monitored the HR until a maximized increase was reached that was close to the baseline HR. Once the HR had been maintained for 10 s without a decrease, we defined the HR as adrenergic HR.

Prevalence of OCR was investigated and patients were divided into two subgroups according to the occurrence of OCR. In addition, prevalence of OCR was compared according to the patient’s diagnosis, and, in patients who underwent two muscle surgeries, also the number and sequence of the operated muscles (first and second operated muscle). Additionally, the prevalence of OCR between the specific operated muscle groups were compared. The surgical factors associated with the occurrence of OCR were analyzed. The changes in HR each stage during the surgery were evaluated and compared between patients with and without OCR.

The data were analyzed using SPSS software version 21.0 (SPSS Inc., Chicago, IL, USA), and the clinical values were compared and analyzed using the Mann-Whitney test and Chi-square test. Multivariate logistic regression was used to analyze the risk factors associated with the occurrence of OCR. A *p*-value ≤0.05 was considered statistically significant.

## Results

A total of 99 patients, with 162 operated muscles, who underwent strabismus surgery, were included in this study. During surgery, OCR occurred in 64 (65%) patients. The mean age was 11.1 ± 8.9 years in patients with OCR, and 7.9 ± 6.8 years in patients without OCR. Male patients accounted for 36 (56%) and 14 (40%) patients in each group, respectively. There were no significant differences in age and gender between patients with and without OCR. In total, 79 patients (80%) were diagnosed with exotropia, 9 patients (9%) with esotropia, and 13 patients (11%) with superior oblique palsy. There were no significant differences of strabismus diagnosis in patients with and without OCR (*p* = 0.28). Basic demographics are presented in Table 1.

**Table 1:** Preoperative demographics of the patients

	OCR (*n* = 64)	without OCR (*n* = 35)	p
Age, years (range)	11.1 ± 8.9(2–52)	7.9 ± 6.8(1–42)	0.07^a^
Male (%)	36(56)	14(40)	0.12^b^
Diagnosis (%)			0.28^b^
Exotropia	54(84)	25(72)	
Esotropia	5(8)	4(11)	
Superior oblique palsy	5(8)	6(17)	

OCR, oculocardiac reflex

^a^Mann-Whitney test

^b^Chi-square test

The mean number of operated muscles was 1.6 ± 0.5 in patients with OCR and 1.7 ± 0.5 in patients without OCR. There was no difference between the groups (*p* = 0.56). Baseline HR was higher in patients without OCR (131.1 ± 16.3 beat/min) than in patients with OCR (122.2 ± 17.5 beat/min) however, there was no statistical difference of HR between 2 groups. Thirty-eight (59%) patients with OCR underwent two muscle surgeries, compared to 22 (63%) patients without OCR. However, there was no significant difference between the groups (*p* = 0.39). The incidence of OCR in muscles undergoing recession, resection and oblique surgery was 70 (41%), 12 (7%) and 4 (3%), respectively (*p* = 0.02). In the surgical techniques, resection surgeries were significantly prevalent in patients with OCR than in patients without OCR (*p* = 0.03).

In the 162 muscles that were operated on, 122 (76%) were lateral rectus; 26 (16%), medial rectus; 2 (1%), superior rectus; and 12 (7%), inferior rectus. There was no significant difference in the occurrence of OCR according to the operated muscle (*p* = 0.36). Table 2 details the surgical information.

**Table 2:** Surgical measurements stratified by the occurrence of oculocardiac reflex

	OCR (n = 64)	without OCR (n = 35)	p
Number of operated muscle (range)	1.6 ± 0.5(1–2)	1.7 ± 0.5(1–2)	0.56^a^
Operated muscle (%)			0.36^b^
Lateral rectus muscle	59(72)	63(79)	
Medial rectus muscle	17(21)	9(11)	
Superior rectus muscle	2(2)	1(1)	
Inferior oblique muscle	4(5)	7(9)	
Baseline HR, beat/min	122.2 ± 17.5(78–162)	131.1 ± 16.3(89–166)	0.06^a^
Two muscle surgery (%)	38(59)	22(63)	0.39^a^
Surgery (%)			
Recession	70(41)	76(43)	0.02^b^
Resection	12(7)	3(2)	
Oblique surgery	4(3)	7(4)	

Recession surgery included the rectus muscle and inferior oblique muscle recession, OCR, oculocardiac reflex; HR, heartrate

^a^Mann-Whitney test

^b^Chi-square test

Sixty (60%) patients underwent two muscle surgeries. In the analysis based on the sequence of the operated muscles, incidence of OCR were significantly higher in the first (*n* = 48, 80%) than the second operated muscle (*n* = 18, 30%) (*p* = 0.01). Additionally, there were 46 (46%) patients with bilateral rectus muscle recession muscle surgery in this study. According to the sequence of operated muscle, the prevalence of OCR was higher in first operated lateral rectus muscle than second lateral rectus muscle (*p* < 0.01) (Table 3 and Table 4).

**Table 3:** The occurrence of oculocardiac reflex according to the sequence of operated muscle in two muscle surgery and bilateral lateral rectus recession surgery

	OCR	without OCR	p^a^
Sequence of operated muscle (%)			< 0.01
First muscle	38(63)	22(37)	
Second muscle	18(30)	42(70)	
Sequence of operated muscle in BLR recession (%)			< 0.01
First lateral rectus muscle	33(72)	13(28)	
Second lateral rectus muscle	16(35)	30(65)	

OCR, oculocardiac reflex; BLR, bilateral lateral rectus muscle

^a^Chi-square test

**Table 4:** The occurrence of oculocardiac reflex and heart rate at each phase in one and two muscle surgery

	One muscle surgery	Two muscle surgery	
		first muscle	second muscle
Patients	39	60	60
OCR (%)	26(67)	38(63)	18(30)
Heart rate, beat/min			
Baseline	125.1 ± 15.9	128.8 ± 17.8	125.1 ± 18.3
Calculated percentage (median, range), %	100	100	100
Traction	89.9 ± 24.4	97.6 ± 24.5	105.7 ± 25.8
Calculated percentage (median, range), %	67.5 ± 12.1 (63.2, 45.1–84.4)	75.1 ± 9.8 (70.4, 38.4–87.6)	83.5 ± 12.2 (83.1,67.7–92.2)
Adrenergic	110.1 ± 19.8	113.1 ± 19.6	117.5 ± 22.1
Calculated percentage (median, range), %	84.1 ± 11.8 (80.2, 68.3–90.2)	89.3 ± 12.4 (85.9,73.2–93.2)	93.4 ± 4.6 (90.3,72.3–98.2)
Cut muscle	114.9 ± 18.9	118.2 ± 19.0	119.8 ± 20.9
Calculated percentage (median, range), %	91.3 ± 9.6 (87.9, 76.4–96.8)	90.7 ± 5.6 (89.4,77.9–98.7)	93.7 ± 3.4 (91.2,78.5–98.2)

OCR, oculocardiac reflex

In multivariate logistic regression analysis, age, gender, and single muscle surgery were not associated with the occurrence of OCR (*p* = 0.08, 0.12 and 0.82). Resection and medial rectus muscle surgeries were highly associated with a greater occurrence of OCR (OR = 3.57 and 3.31, respectively); however, they were not significantly correlated (*p* = 0.06 and 0.11, Table 5). Additionally, the first operated EOM at surgery was significantly associated with the occurrence of OCR (OR = 3.95, *p* < 0.01, Table 5).

**Table 5:** Multivariate logistic regression analysis for the occurrence of oculocardiac reflex

Variable	OR	95% CI		p
Age	1.07	0.99	1.15	0.08
Male	0.52	0.22	1.19	0.12
Baseline HR	0.97	0.95	0.99	0.09
Resection	3.57	0.94	13.47	0.06
Medial rectus muscle	3.31	0.76	14.38	0.11
Single muscle surgery	0.91	0.39	2.12	0.82
First operated EOM	3.95	1.85	8.41	< 0.01

HR, heartrate; EOM, extraocular muscle; OR, odds ratio; CI, confidential interval

## Discussion

The OCR is a common occurrence in strabismus surgery. The incidence of OCR reported in previous studies is greatly varied and depends on the method of evaluation and definition of OCR. In this study, the incidence of OCR, which was defined as the maximum decrease in HR at the first traction of the muscle (≥ 20% reduction from baseline HR) was 65%. This is similar to incidences reported in other studies [11, 12, 15].

Several risk factors, such as the patient’s age and gender, use of premedication, anesthetic technique, type of surgery, and the sequence of the operated muscle, have been shown to influence the occurrence of OCR. A number of studies have shown that the occurrence of OCR was higher in female patients, however, there are studies that have reported no difference between the gender of patients with OCR [11, 12]. There was no gender difference between patients with and without OCR in this study. An intravenous anticholinergic agent was used as a premedication in pediatric surgeries, therefore, we could not conclude on a possible preventive effect of premedication on OCR. Additionally, the surgical method and number of operated muscles were not associated with the occurrence of OCR in this study.

The results of this study show that OCR was more prevalent in the resection of EOM; however, no significant association between specific muscles and OCR was found. Previous studies have reported that medial rectus surgery is strongly associated with OCR [11, 12, 15]. However, medial rectus muscle surgery was not significant risk factor for OCR in this study. In this study, most of the operated muscles were lateral rectus muscles unilaterally or bilaterally rather than other muscles, due to the high prevalence of exotropia in Korea, when compared to Western countries. Additionally, most of resection surgeries were performed to medial rectus muscle with exotropia. Clinically, it speculated that the traction of the medial rectus muscle might trigger OCR in resection surgery, however, our study did not show support that the surgery on the medial rectus muscle is a risk factor for oculocardiac reflex.

Also, we did not find a statistical difference in the occurrence of OCR between patients with one or two muscle surgeries. However, there was a higher prevalence of OCR in first operated muscle among patients having two muscle surgeries. The first operated muscle was significantly associated with the occurrence of OCR in this study. This is in agreement with Lai et al., who reported that the occurrence of OCR was significantly higher during traction of the first operated muscle [12]. We speculated that the first operated muscle might be more likely to trigger OCR regardless of specific type of EOM. Additionally, we demonstrate that the prevalence of OCR was high in first operated muscle, even though same muscles in both eye although we could not explain this phenomenon exactly.

When OCR occurred after first mechanical stimulation of the EOM, the counter-regulatory process could lead to the adaption of a subsequent stimulus, due to the occurrence of OCR. We speculated that this process could affect the infrequency of OCR in the second operated muscle. Thus, the first operated muscle, with multiple surgeries, might be vulnerable to OCR, regardless of the specific muscle or surgical technique. Machida et al. reported that uniformed and objective EOM tension without atropine as premedication and recovery for OCR were evaluated at the strabismus surgery [13].

Braun et al. first reported the adrenergic phase of OCR in 1992 [14]. It described the counter-regulatory effect that outlasted the period of mechanical stimulation. This phenomenon occurred through the spontaneous recovery of HR after traction of the EOM and is referred to as the ‘adrenergic phase’ or ‘vagal escape’ of the OCR [6, 14]. In this study, the maximum recovered HR after traction of the EOM was measured, and interestingly, it did not fully recover until the cutting of the EOM, during surgery in patients with OCR. The mean percentage of the decreased HR, when compared to baseline HR, was 10% at the cutting of the EOM in patients with OCR. We speculated that when the OCR first occurred, it would be relatively difficult for the HR to fully recover to the baseline HR at any point in the surgery. Therefore, it might be important for the surgeon to monitor the occurrence of OCR at the first traction of the EOM.

This study has some limitations. Firstly, the design of this study was retrospective. Secondly, the patients in this study underwent surgery using an anticholinergic agent as a premedication. Thus, the occurrence of OCR could be affected by this pharmacologic agent. However, the use of an anticholinergic agent is still controversial in the prevention of OCR. Secondly, the majority of patients underwent lateral rectus muscles for exotropia. Therefore, there was an unbalanced distribution of operated muscles. As exotropia, such as intermittent exotropia, is common in East Asia, recession of the lateral rectus muscle is a more common procedure, when compared to other EOMs. Lastly, we did not measure objective information of traction muscles such as duration, amount of force. In this study, we hooked the muscle persistently until observing maximal recovery of decreased heart rate comparing with baseline heart rate. Thus, the duration of tension was variable at each surgical cases. These variability of traction could affect incidence of OCR during surgery. We performed the surgery, routinely without the manipulation of traction force.

## Conclusions

A reduction in HR at the traction of the muscle during surgery did not fully recover in patients with OCR. The first operated muscle in patients with two muscle surgeries was a significant risk factor for OCR occurrence. Surgeon needs to be more careful while operating first extraocular muscle in strabismus surgery. The occurrence of OCR may be an important surgical consideration for patients with strabismus.

## Abbreviations

EOM: Extraocular muscle
HR: Heart rate
OCR: Oculocardiac reflex
